# Acute Hyperglycemia Does Not Impair Microvascular Reactivity and Endothelial Function during Hyperinsulinemic Isoglycemic and Hyperglycemic Clamp in Type 1 Diabetic Patients

**DOI:** 10.1155/2012/851487

**Published:** 2012-01-04

**Authors:** Eva Horová, Jiří Mazoch, Jiřina HiIgertová, Jan Kvasnička, Jan Škrha, Jan Šoupal, Martin Prázný

**Affiliations:** ^1^3rd Department of Internal Medicine, 1st Faculty of Medicine, Charles University, U Nemocnice 1, 12800 Prague, Czech Republic; ^2^Department of Hematology, 1st Faculty of Medicine, Charles University, U Nemocnice 2, 12800 Prague, Czech Republic

## Abstract

*Aims*. The aim of this study was to evaluate the effect of acute glycemia increase on microvasculature and endothelium in Type 1 diabetes during hyperinsulinemic clamp. *Patients and Methods*. Sixteen patients (51 ± 7 yrs) without complications were examined during iso- and hyperglycemic clamp (glucose increase 5.5 mmol·L^−1^). Insulin, lipid parameters, cell adhesion molecules and fibrinogen were analyzed. Microvascular reactivity (MVR) was measured by laser Doppler flowmetry. *Results*. Maximum perfusion and the velocity of perfusion increase during PORH were higher in hyperglycemia compared to baseline (47 ± 16 versus 40 ± 16 PU, *P* < 0.01, and 10.4 ± 16.5 versus 2.6 ± 1.5 PU·s^−1^, *P* < 0.05, resp.). Time to the maximum perfusion during TH was shorter and velocity of perfusion increase during TH higher at hyperglycemia compared to isoglycemic phase (69 ± 15 versus 77 ± 16 s, *P* < 0.05, and 1.4 ± 0.8 versus 1.2 ± 0.7 PU·s^−1^, *P* < 0.05, resp.). An inverse relationship was found between insulinemia and the time to maximum perfusion during PORH (*r* = −0.70, *P* = 0.007). *Conclusion*. Acute glycemia did not impair microvascular reactivity in this clamp study in Type 1 diabetic patients. Our results suggest that insulin may play a significant role in the regulation of microvascular perfusion in patients with Type 1 diabetes through its vasodilation effect and can counteract the effect of acute glucose fluctuations.

## 1. Introduction

Hyperglycemia is an independent risk factor for the development of vascular complications in patients with diabetes. It has multiple negative effects on vessel wall, and maintaining good glycemic control significantly reduces the risk of both microvascular and macrovascular complications in diabetic patients [1–4]. Short-term glycemic variability is also discussed as a potential additional independent risk factor for the development of diabetic complications. Increased glycemic variability may contribute to the risk of micro- and macrovascular complications in patients with Type 2 diabetes [5–8]. One solitary study on a smaller number of Type 1 diabetic patients indicates a possible connection between increased glycemic variability and the development of diabetic neuropathy [9]. However, based on the analyses of data from DCCT study, it was reported that increased glycemic variability is not an additional factor contributing to the risk of microvascular complications in Type 1 diabetic patients [10, 11]. As the data regarding the effect of increased glycemic variability on diabetic complications are limited, the importance of glycemic variability in Type 1 diabetes is not yet clear.

It is also not quite clear how increased glycemic variability could induce diabetic complications. Oxidative stress was suggested as a possible common mechanism of the endothelial impairment induced by increased glycemic variability [12–16]. Endothelial dysfunction, a first event in the process of vascular impairment, can be induced by increased oxidative stress. It has been shown in experimental studies in Type 2 diabetic patients that both single and multiple glycemia increase induce oxidative stress and impair endothelial function [17, 18]. Again, especially in Type 1 diabetic patients, the data on the effects of acute hyperglycemia on vascular function are limited.

In Type 2 diabetes, hyperglycemia is often associated with hyperinsulinemia (depending on the stage of diabetes), dyslipidemia, arterial hypertension, and other metabolic abnormalities that may modify the impact of acute hyperglycemia on vascular function [19, 20]. Although the metabolic disorders typically associated with Type 2 diabetes are usually not present in Type 1 diabetes, insulin alters the vascular function in Type 1 diabetes as well [21]. Both hyperglycemia and hyperinsulinemia can affect vascular reactivity [21, 22], and it is even not clear whether some abnormalities in vascular function cannot be attributed to hyperinsulinemia rather than to hyperglycemia [23].

It has been shown either in vitro or in vivo that insulin exerts an anti-inflammatory and/or antiprooxidant action [24, 25]. The recent work of Monnier et al. [26] indicates significant differences between Type 1, insulin-treated, and non-insulin-treated Type 2 diabetic patients in the activation of oxidative stress and its association with insulin treatment and glycemic variability. These differences may be partially explained by the beneficial effect of insulin on the activation of oxidative stress.

The objective of this study was to evaluate the effects of acute glycemia increase on microvascular reactivity and endothelial function in Type 1 diabetic patients during isoglycemic and hyperglycemic hyperinsulinemic clamp. Possible relationship between rapidly induced hyperglycemia and oxidative stress was analyzed. The rationale for this study is a limited number of studies regarding glycemic variability performed so far in Type 1 diabetic patients, unclear effect of single rapid glycemia increase in Type 1 diabetic patients, and insufficient understanding of differences in the effect of glycemic variability and insulin action on diabetic complications in patients with Type 1 and 2 diabetes who represent two groups with different metabolic and treatment profiles. 

## 2. Materials and Methods

### 2.1. Patients

Sixteen patients with type 1 diabetes (mean age 51 ± 7 years, mean BMI 25.7 ± 3.7 kg·m^−2^, mean diabetes duration 24 ± 7 years, and mean HbA_1C_    7.8 ± 0.9% according to IFCC calibration) were studied. The diagnosis of diabetes was based on its clinical appearance and undetectable C-peptide levels (mean serum C-peptide < 0,05 nmol/l). Only patients without macro- and microvascular complications were included in the study. All patients were treated with continuous subcutaneous insulin infusion (CSII) using insulin pump in basal-bolus regimen. Insulin used was either insulin analog lispro (*n* = 8) or aspart (*n* = 8). The mean total daily basal dose was 24.9 ± 9.1 U, mean total daily bolus dose was 21.8 ± 5.7 U, and the mean total daily dose of insulin in studied subject was 47.0 ± 14 U. Study was approved by the local ethics committee and all subjects gave their written informed consent before the experiment.

### 2.2. Clamp and the Control Test

Two tests were done in this study: standard iso- and hyperglycemic clamp was performed first and then in 2–8 weeks the patients underwent an isoglycemic control test. The control test without insulin infusion and glucose increase was performed to test for non-specific effects of volume load (intravascular volume expansion, hemodilution), so the patients served also as the control group for themselves. Two of the sixteen patients refused to participate in the control test. Clamp test was performed by the method described previously [27, 28] using intravenous human regular insulin infusion rate 0.001 IU·kg^−1^·min^−1^. Insulin pump was stopped immediately before the start of the test and started again after the end of the test. Baseline glucose was 6.8 ± 2.7 mmol·L^−1^. Isoglycemic phase of the clamp was maintained for 3 hours (mean venous blood glucose 6.5 ± 1.9 mmol·L^−1^) and consequent hyperglycemic phase for next 3 hours (mean venous blood glucose 12.0 ± 1.6 mmol·L^−1^). Venous blood glucose was measured in 5–10 min intervals using Super GL ambulance analyzer (Freital, Germany). Glucose 20% solution was infused at variable rate depending on venous blood glucose level. Parameters of insulin sensitivity were calculated: glucose disposal rate (M), metabolic clearance of glucose (MCR_G_), and insulin sensitivity index (MCR_G_·I^−1^).

During the control test, only intravenous solutions (0.9% natrium chloride solution and 20% glucose solution) of the same volume as in the clamp test were infused to each patient during 6 hours. Insulin pump was running at the patient's usual insulin basal rate, and glycemia was maintained at the baseline value using variable rate of 20% glucose infusion. Baseline venous blood glucose was 6.9 ± 2.2 mmol·L^−1^ and glycemia at the end of the test was 6.9 ± 1.8 mmol·L^−1^.

### 2.3. Biochemical Methods

Plasma insulin concentrations were measured at the baseline, isoglycemic, and hyperglycemic phase of the clamp test and at the baseline and the end of the control test. Insulin was measured by radioimmunoassay kits (CIS Bio International, France), with 5.6% intraassay and 7.2% interassay variabilities (CV) in our laboratory. Other biochemical parameters were measured at the baseline and at the end of the clamp and the control test. Total serum cholesterol (TC), HDL-cholesterol (HDL-C), and triglycerides (TG) were measured by photometric enzymatic method on an automated analyzer (COBAS Mira, Roche). LDL-cholesterol (LDL-C) was calculated using the friedwald formula. Serum concentrations of cell adhesion molecules E-selectin, P-selectin, intercellular cell adhesion molecule 1 (ICAM-1), and vascular cell adhesion molecule (VCAM) were measured with ELISA kits manufactured by RD system Europe (Abingdon, UK). The immunoassays had an intra-assay variability below 5% and an inter-assay variability below 8%. All samples from the patients were measured in one assay to minimize the effect of variation. Plasma malonyl dialdehyde concentration (MDA) was measured using fluorimetric method [29] with intra-assay variability below 3% and inter-assay variability below 7% in our laboratory. Fibrinogen was measured by the clauss method [30] on an automatic analyzer.

### 2.4. Laser Doppler Flowmetry

Skin microvascular reactivity (MVR) was measured at the baseline and at the end of isoglycemic and hyperglycemic clamp phase and at the baseline and at the end of the control test. MVR was measured by laser Doppler flowmetry using a PeriFlux PF 4001 Master laser instrument and a PeriTemp 4001 Heater thermostatic unit manufactured by Perimed (Sweden). Instrument settings were as follows: time constant 0.02 s, sampling frequency 32 Hz, averaging from two samples. Measurements were done at a room temperature of 22°C. Postocclusive reactive hyperemia (PORH) and thermal hyperemia (TH) tests were used for the assessment of microvascular reactivity. Measurement was done in two locations, fingertip (fi) and forearm (fo). One standard probe (type 408, fibre separation 0.25 mm) was attached to the fingertip of the third finger of the nondominant upper extremity to record PORH only. Second thermostatic probe (type 455, 23 mm diameter, fiber separation 0.25 mm) was used for the recording of PORH and TH on forearm. Optical fibers in this probe are integrated into the heating plate and thus the entire area of tissue under the probe is heated. The probe was fixed with double-stick discs (3 M, USA) to the skin of forearm and its temperature was set to 32°C for the purpose of skin thermal stabilization during PORH and before TH. A temperature of 44°C was used during TH as the thermal stimulus. Basal perfusion (PORHb) was measured for 2 min before the PORH test. The brachial artery was then occluded by a sphygmomanometer cuff inflated to a suprasystolic pressure for 3 minutes and PORH was recorded after its sudden release. Maximal perfusion during hyperemia was recorded (PORHmax) as well as the time needed for reaching this maximal perfusion (PORHtmax). The velocity of the perfusion increase (PORHmax/t) was calculated as the ratio of (PORHmax-PORHb) and PORHtmax. Thermal hyperemia was measured 10 minutes after the PORH test. The probe temperature was set to 44°C, and parameters THmax, THtmax, and THmax/t were recorded or calculated similarly as those in the PORH test. Perfusion is given in arbitrary perfusion units (PU). Perisoft for Windows 2.5 software was used for recording and evaluating data. Records were blinded and the evaluation was performed by a single operator.

Statistical evaluation was performed by Statistica for Windows software. Basic descriptive statistics were calculated for presented parameters. ANOVA, Student's *t-*test or Wilcoxon's test, Mann-Whitney, and Kolmogorov-Smirnov tests were used for comparing data. Pearson's and Spearman's correlations were used for analysis of relationships between measured parameters. Data are expressed as mean ± S.D.

## 3. Results

Results of insulin sensitivity, lipid parameters, fibrinogen, oxidative stress, cell adhesion molecules, and microvascular reactivity are presented in Table 1. Baseline values of all variables measured in the clamp and control test were comparable, no statistically significant differences were found.

Serum total, HDL, and LDL-cholesterol decreased significantly in the hyperglycemic phase of the clamp compared to the baseline, and similarly their concentration decreased at the end of the control test. Serum triglycerides and MDA concentrations did not change significantly. Concentrations of cell adhesion molecules (E- and P-selectin, ICAM-1 and VCAM) decreased significantly in the hyperglycemic phase of the clamp as compared to the baseline, and similarly they decreased during the control test (except for P-selectin during the control test where only a trend to decrease could be observed).

### 3.1. MVR at the Baseline of the Clamp

**Figure 1:**
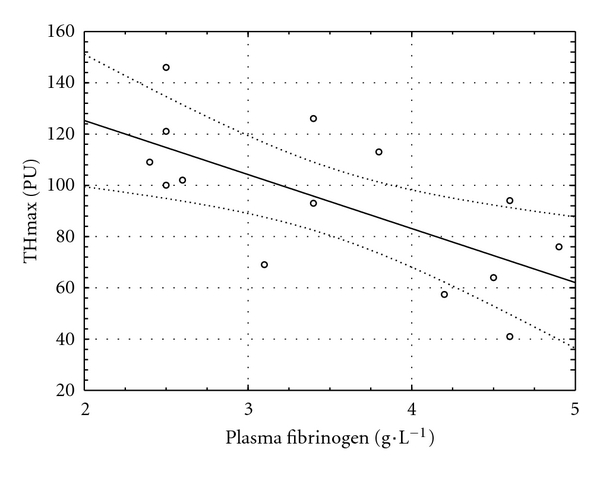
Relationship between plasma fibrinogen concentration and maximal perfusion during thermal hyperemia (THmax) at the baseline of the clamp (*y* = 167.44 − 21.07 × *x*; *r* = −0.67; *P* = 0.009).

**Figure 2:**
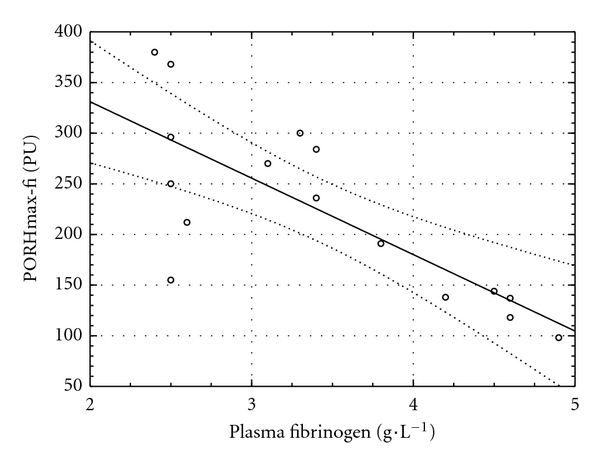
Relationship between plasma fibrinogen concentration and maximal perfusion during postocclusive reactive hyperemia at the fingertip (PORHmax-fi) at the baseline of the clamp (*y* = 482.04 − 75.47 × *x*; *r* = −0.77; *P* = 0.0005).

**Figure 3:**
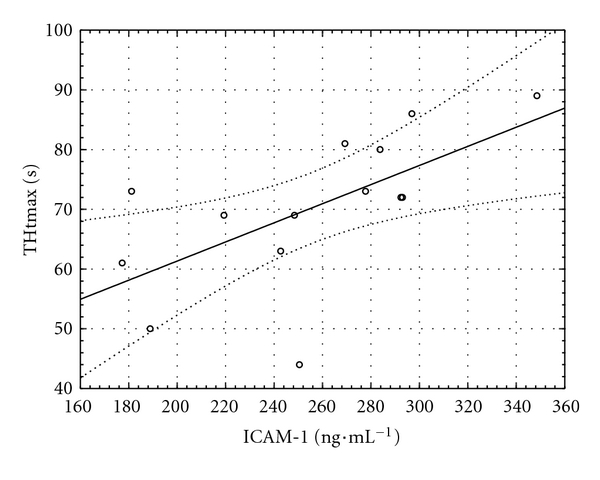
Relationship between serum intercellular cell adhesion molecule-1 (ICAM-1) and the time to maximal perfusion during thermal hyperemia (THtmax) at the baseline of the clamp (*y* = 29.34 + 0.16 × *x*; *r* = −0.63; *P* = 0.016).

A negative relationship was found between fibrinogen concentration and maximal perfusion in thermal hyperemia and post-occlusive reactive hyperemia at the fingertip (*r* = −0.67, *P* = 0.009, Figure 1, and  *r* = −0.77, *P* < 0.0005, Figure 2, resp.). Another relationship was observed between ICAM-1 and time to maximum perfusion during TH (*r* = 0.63, *P* = 0.016, Figure 3).

### 3.2. MVR at the Isoglycemic Phase of the Clamp

In the forearm, time to reach the maximum perfusion during PORH was significantly shorter and the velocity of perfusion increase during PORH was higher in the isoglycemic clamp phase compared to baseline (Table 1). Parameters of microvascular reactivity measured at the fingertip did not change significantly.

### 3.3. MVR at the Hyperglycemic Phase of the Clamp

At the forearm, maximum perfusion and the velocity of perfusion increase during PORH were significantly higher in the hyperglycemic phase of the clamp compared to baseline. In thermal hyperemia, no changes of MVR were observed in the clamp compared to baseline. However, time to reach the maximum perfusion during TH was shorter and velocity of perfusion increase during TH was higher at the hyperglycemic phase compared to the isoglycemic phase. Parameters of microvascular reactivity measured at the fingertip did not change significantly.

**Figure 4:**
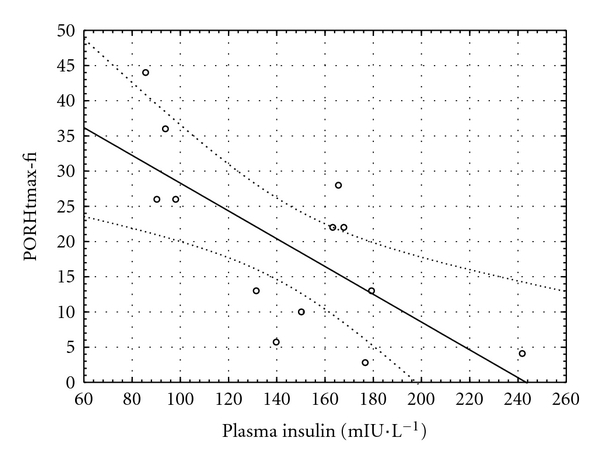
Relationship between plasma insulin concentration and the time to maximal perfusion during postocclusive hyperemia at the fingertip (PORHtmax-fi) in the hyperglycemic phase of the clamp (*y* = 48.03 −  0.20 × *x*; *r* = −0.70; *P* = 0.007).

**Figure 5:**
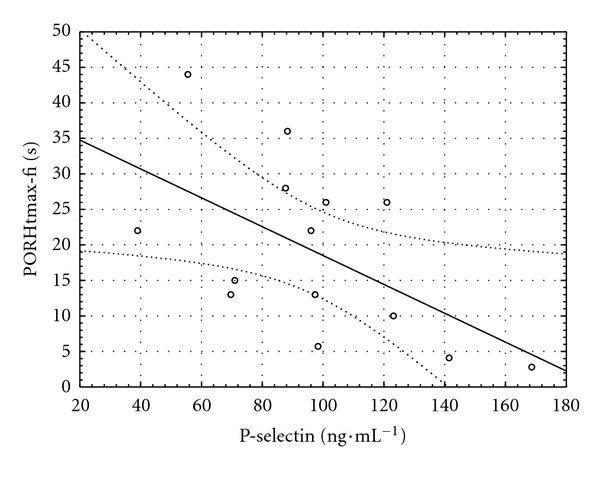
Relationship between serum P-selectin concentration and the time to maximal perfusion during postocclusive reactive hyperemia at the fingertip (PORHtmax-fi) in the hyperglycemic phase of the clamp (*y* = 38.83 − 0.20 × *x*; *r* = −0.57; *P* = 0.035).

**Figure 6:**
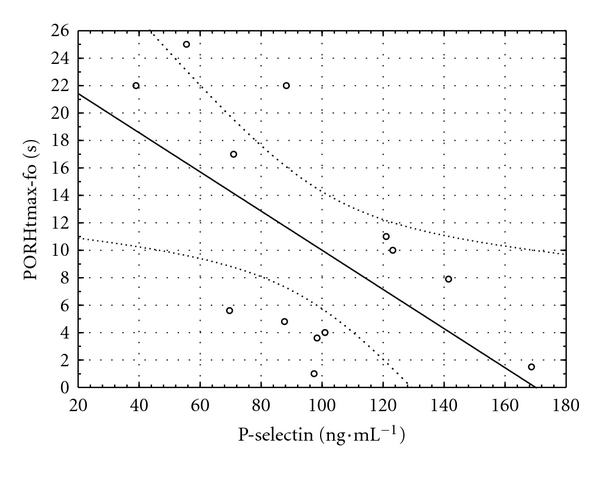
Relationship between serum P-selectin concentration and the time to maximal perfusion during postocclusive reactive hyperemia in the forearm (PORHtmax-fo) in the hyperglycemic phase of the clamp (*y* = 24.21 − 0.14 × *x*; *r* = −0.60; *P* = 0.029).

An inverse relationship was found between insulinemia and the time to maximum perfusion during PORH at the fingertip (*r* = −0.70, *P* = 0.007, Figure 4). Another inverse relationship was observed between P-selectin and time to maximum perfusion during PORH both at fingertip and forearm (*r* = −0.57,  *P* = 0.035, and *r* = −0.60,  *P* = 0.029, Figures 5 and 6, resp.).

 No relationship was observed between venous blood glucose and MVR at any point during the clamp and the control test. Similarly, no relationship was observed between parameters of insulin sensitivity and MVR.

### 3.4. MVR in the Control Test

During the control test, no changes in MVR were observed at all, both for PORH and TH tests.

## 4. Discussion

In this study, we did not prove the hypothesis that rapidly induced hyperglycemia impairs microvascular reactivity in patients with Type 1 diabetes. No relationship was observed between venous glucose and microvascular reactivity during the hyperinsulinemic isoglycemic and hyperglycemic clamp. This is in accordance with the findings of Oomen et al. [22] in a similar study.

Dynamic parameters of MVR indicated faster microvascular vasodilation in the isoglycemic phase during which only hyperinsulinemia was present. Faster microvascular vasodilation persisted also in the hyperglycemic phase and was not abolished by the rapid glycemia increase. In the hyperglycemic phase, insulinemia was associated with the time needed to reach the maximum perfusion during PORH at the fingertip resulting in faster onset of the maximum perfusion in patients with higher insulin concentration. Our results suggest that the vasodilation effect of insulin may play a significant role in the regulation of microvascular perfusion in patients with diabetes. The reason why we did not observe significant correlation between insulinemia and parameters of MVR also in forearm resides possibly in capillary bed differences. Capillary bed of the fingertip is different from forearm [31]. It is more dense and contains multiple shunts. It is considered rather functional than simply nutritional. The basal perfusion is usually several times higher in fingertips than in other skin locations. It may be therefore easier to detect the vasodilation related to insulin in fingertip than in forearm where we observed only nonsignificant correlations between insulinemia and MVR during the clamp.

Chronic hyperglycemia induces biochemical abnormalities in many systems including hemocoagulation/fibrinolysis pathways and cell adhesion molecules and induce oxidative stress [32–35]. However, we did not observe any alteration in these systems following the clamp, probably a single and relatively short clamp test was not enough to induce significant changes [36]. Significant decline in cell adhesion molecules concentration during the clamp (as well as in lipid parameters) was probably nonspecific as it was reproduced in the control test as well. It was probably caused by hemodilution during relatively high intravenous volume load. The control test was included in the study protocol design to exclude the influence of any non-specific effects, especially for the effect of intravenous volume load. Infusion of intravenous fluids in larger quantities is necessarily connected with clamp techniques. In the control test, we have not observed any changes in microvascular reactivity in the link with increased intravascular volume.

Hyperglycemia induces platelet aggregation and activates procoagulation factors not only in Type 2 diabetic patients [37], but also in healthy subjects [38, 39], and coagulation is closely related to endothelial function. This is in an accordance with our observation of a negative relationship between plasma fibrinogen concentration and MVR at the baseline of the clamp. Surprisingly, higher levels of P-selectin, a marker of platelet activation and procoagulation [40], were associated with faster vasodilation in PORH both at fingertip and in the forearm at the end of the clamp test. Unfortunately, it is not possible to distinguish whether this observation is related to hyperglycemia, hyperinsulinemia, or both.

The influence of insulin on the development of vascular complications in diabetes is still unclear. Insulin concentration varies in different types of diabetes (depending on the type and progress of metabolic disorder) and is often massively modified by antidiabetic treatment. This further complicates the understanding of relationship between insulin levels and vascular complications. Insulin levels are usually higher in early stages of Type 2 diabetes (or even in prediabetes) and decline consequently [41]. Insulin deficiency develops in a large portion of Type 2 diabetic patients due to the apoptosis of B cells [42]. As an example of a study designed to eliminate the effect of insulin, Ceriello et al. described a more deleterious effect of fluctuating glycemia than that of sustained hyperglycemia on oxidative stress and endothelial function in Type 2 diabetic patients whose insulin secretion was completely blocked by somatostatin during the experiments [17]. On the other hand, Monnier et al. described similar findings in the presence of insulin in a clinical setting using continual glucose monitoring system in otherwise normally treated Type 2 diabetic patients [18]. Further data published recently by Monnier et al. indicate that the activation of the oxidative stress as assessed from 24 h urinary excretion rates of 8-isoPGF2alpha is within the normal range in all diabetic patients treated with insulin (both Type 1 and Type 2) whilst this parameter is significantly elevated in those patients who use oral hypoglycemic agents and no insulin treatment [26]. In the same study, an association between the activation of oxidative stress and glycemic variability was found only in the group of non-insulin-treated Type 2 diabetic patients. Such results are in agreement with those found in the present work and seem to indicate that insulin per se exerts beneficial effect on several factors that may play a role in the development of diabetic complications.

In Type 1 diabetic patients, diabetes is manifested when decline in B cell leads to clinically significant insulin deficiency. Following the initiation of insulin substitution therapy in these patients, they can have hypo-, normo- or even hyperinsulinemia depending on the dose of injected or infused insulin [43]. Concentration of glucose fluctuates in these patients (as well as in insulin-treated Type 2 diabetic patient) according to insulin dose. When glycemic variability is considered a risk factor for the development of vascular complications by some authors, a question shall be raised if even insulin variability cannot participate as well. In studies testing vascular function in diabetic patients, it may be therefore very useful to measure and mention the insulin levels despite some technical difficulties and always check for possible relationship between insulin concentration and vascular function.

## 5. Conclusion

In the isoglycemic and hyperglycemic hyperinsulinemic clamp, we have not observed any impairment of microvascular reactivity following fast glycemia increase in patients with Type 1 diabetes. Our results suggest that insulin may play a significant role in the regulation of microvascular perfusion in patients with diabetes through its vasodilation effect. We have also shown the importance of the control test with intravenous volume load in studies using clamp techniques. Further research is necessary to clarify the effect of glucose and insulin fluctuation on vascular function and development of diabetic complications.

## Figures and Tables

**Table 1:** Biochemical parameters, parameters of insulin sensitivity and microvascular reactivity during isoglycemic (ISO) and hyperglycemic (HYPER) hyperinsulinemic clamp and during the control test in Type 1 diabetic patients.

	Clamp (*n* = 16)			Control test (*n* = 14)	
	Baseline	ISO	Hyper	Baseline	End
Glucose (mmol·L^−1^)	6.8 ± 2.7	6.5 ± 1.9	12.0 ± 1.6	6.9 ± 2.2	6.9 ± 1.8
Insulin (mIU·L^−1^)	55 ± 39	155 ± 54^a^	152 ± 47^a^	74 ± 55	65 ± 46
M (*μ*mol/kg·min)	—	30 ± 10	57 ± 26	—	—
MCR_G_ (mL/kg·min)	—	5.0 ± 2.3	4.8 ± 2.4	—	—
MCR_G_/L (mL/kg·min per mU/L ×100)	—	3.5 ± 1.6	3.5 ± 2.2	—	—

TC (mmol·L^−1^)	4.6 ± 0.7	—	4.3 ± 0.6^b^	4.7 ± 0.8	4.3 ± 0.8^b^
HDL-C (mmol·L^−1^)	1.3 ± 0.4	—	1.2 ± 0.4^b^	1.3 ± 0.4	1.2 ± 0.3^b^
LDL-C (mmol·L^−1^)	2.9 ± 0.6	—	2.7 ± 0.5^b^	2.9 ± 0.6	2.7 ± 0.6^b^
TG (mmol·L^−1^)	0.9 ± 0.3	—	0.8 ± 0.2	1.1 ± 0.7	1.0 ± 0.5

Fibrinogen (g· L^−1^)	3.4 ± 0.9	—	3.3 ± 0.8	3.4 ± 1.0	3.1 ± 0.9^b^
MDA (*μ*mol·L^−1^)	1.8 ± 0.3	—	1.8 ± 0.2	1.8 ± 0.5	1.7 ± 0.3

E-selectin (ng·mL^−1^)	29 ± 12	—	27 ± 11^a^	29 ± 15	28 ± 14^b^
P-selectin (ng·mL^−1^)	132 ± 50	—	93 ± 34^a^	126 ± 50	111 ± 27
ICAM-1 (ng·mL^−1^)	251 ± 48	—	215 ± 41^a^	237 ± 40	212 ± 33^b^
VCAM (ng·mL^−1^)	812 ± 183	—	673 ± 117^a^	815 ± 203	665 ± 165^a^

PORHmax-fi (PU)	224 ± 88	255 ± 112	234 ± 99	212 ± 92	199 ± 60
PORHtmax-fi (s)	24.7 ± 22.1	15.4 ± 11.2	19.1 ± 12.2	19.4 ± 20.3	28.3 ± 27.8
PORHmax/t-fi (PU·s^−1^)	8.3 ± 11.8	9.3 ± 12.6	4.5 ± 4.4	6.7 ± 7.7	13.1 ± 20.5
PORHmax-fo (PU)	40 ± 16	39 ± 19	47 ± 16^b^	32 ± 12	35 ± 12
PORHtmax-fo (s)	13.5 ± 5.1	6.1 ± 4.7^a^	10.4 ± 8.4	11.8 ± 6.0	11.3 ± 9.0
PORHmax/t-fo (PU·s^−1^)	2.6 ± 1.5	11.2 ± 14.9^c^	10.4 ± 16.5^c^	2.7 ± 2.4	3.7 ± 3.0
THmax-fo (PU)	94 ± 29	90 ± 42	97 ± 52	89 ± 46	91 ± 45
THtmax-fo (s)	70 ± 13	77 ± 16	69 ± 15^x^	70 ± 16	64 ± 19
THmax/t-fo (PU·s^−1^)	1.3 ± 0.5	1.2 ± 0.7	1.4 ± 0.8^x^	1.3 ± 1.0	1.7 ± 1.6

Statistical significance of differences as compared to clamp and control test baseline values: ^a^ *P* < 0.001, ^b^ *P* < 0.01, ^c^ *P* < 0.05, and ^x^ *P* < 0.05 when comparing isoglycemic and hyperglycemic phase.

M: glucose disposal rate, MCR_G_: metabolic clearance of glucose, MCR_G_/I: insulin sensitivity index, TC: total cholesterol, HDL-C: HDL-cholesterol, LDL-C: LDL-cholesterol, TG: triglycerides, MDA: malonyl dialdehyde, ICAM-1: intercellular cell adhesion molecule 1, VCAM: vascular cell adhesion molecule, PORHmax-fi: maximal perfusion during postocclusive reactive hyperemia at fingertip, PORHtmax-fi: time to maximal perfusion during postocclusive reactive hyperemia at fingertip, PORHmax/t-fi: velocity of perfusion increase during post-occlusive reactive hyperemia at fingertip, PORHmax-fo: maximal perfusion during post-occlusive reactive hyperemia in forearm, PORHtmax-fo: time maximal perfusion during post-occlusive reactive hyperemia in forearm, PORHmax/t-fo: velocity of perfusion increase during post-occlusive reactive hyperemia in forearm, THmax-fo: maximal perfusion during thermal hyperemia, THtmax-fo: time to maximal perfusion during thermal hyperemia, and THmax/t-fo: velocity of perfusion increase during thermal hyperemia.
